# A software tool for pure‑tone audiometry: Classification of audiograms for inclusion of patients in clinical trials. English version

**DOI:** 10.1007/s00106-015-0089-3

**Published:** 2015-11-25

**Authors:** T. Rahne, F. Buthut, S. Plößl, S. K. Plontke

**Affiliations:** Department of Otorhinolaryngology, University Hospital Halle (Saale), Ernst-Grube-Str. 40, 06120 Halle (Saale), Germany

**Keywords:** Clinical trial, Inclusion criteria, Sudden hearing loss, Software tool

## Abstract

**Objective:**

Selecting subjects for clinical trials on hearing loss therapies relies on the patient meeting the audiological inclusion criteria. In studies on the treatment of idiopathic sudden sensorineural hearing loss, the patient’s acute audiogram is usually compared with a previous audiogram, the audiogram of the non-affected ear, or a normal audiogram according to an ISO standard. Generally, many more patients are screened than actually fulfill the particular inclusion criteria. The inclusion criteria often require a calculation of pure-tone averages, selection of the most affected frequencies, and calculation of hearing loss differences.

**Materials and methods:**

A software tool was developed to simplify and accelerate this inclusion procedure for investigators to estimate the possible recruitment rate during the planning phase of a clinical trial and during the actual study. This tool is Microsoft Excel-based and easy to modify to meet the particular inclusion criteria of a specific clinical trial. The tool was retrospectively evaluated on 100 patients with acute hearing loss comparing the times for classifying automatically and manually. The study sample comprised 100 patients with idiopathic sudden sensorineural hearing loss.

**Results and conclusion:**

The age- and sex-related normative audiogram was calculated automatically by the tool and the hearing impairment was graded. The estimated recruitment rate of our sample was quickly calculated. Information about meeting the inclusion criteria was provided instantaneously. A significant reduction of 30 % in the time required for classifying (30 s per patient) was observed.

**Supplementary file:**

Additional material to this article (Rahne_InclusionCriteria_v.en2.1.xlsx) will be available online at 10.1007/s00106-015-0089-3

Patients included in clinical trials must reliably meet the respective inclusion criteria. Many clinical trials, especially for acute disorders [e. g., idiopathic sudden sensorineural hearing loss (ISSHL)], occur within a busy clinical practice; therefore, the screening of inclusion criteria should be easy and reliable for the investigator. In addition, when planning clinical trials, it is necessary to make accurate recruitment estimates based on retrospective data.

Primary outcomes in clinical trials on treatments of ISSHL are mainly based on average pure-tone thresholds [12, 14, 16, 18, 22]. Owing to the natural course of the disease and the biometrical aspects of the study design, the inclusion and outcome parameters are very heterogeneous [2, 6, 14].

The severity of hearing impairment is quantitatively graded using categories, e. g., “no,” “slight,” “moderate,” “severe,” and “profound” as suggested by the World Health Organization (WHO) [17, 23], but the threshold levels defining those categories vary [17]. The WHO defines “severe impairment” as pure-tone thresholds of the better ear of 61–80 dB HL, whereas the European Working Group on Genetics of Hearing Impairment refers to thresholds of 70–94 dB HL [10]. The calculation of the mean hearing threshold is often based on a four-frequency pure-tone average (4PTA), e. g., of the frequencies 0.5, 1, 2, and 4 kHz [15, 16]; however, a wide variety of outcome parameters have been used in ISSHL studies [14].

Not all frequencies are affected per se in ISSHL. When studying the effects of an intervention, it is meaningful to look at those parameters or frequencies that have been affected by the diseases. An average of all frequencies in a certain predefined region underestimates the effect of an intervention. Therefore, several authors used the three most affected frequencies as primary or secondary outcome measures for evaluation of the treatment [1, 8, 12, 19]. In these cases, the three most affected consecutive frequencies are selected and the hearing loss is calculated at the screening visit relative to a baseline value. There are different options for baseline reference thresholds. The best option for inclusion and/or the outcome measurement would be an audiogram of the affected ear not too long before the incident. However, since such an audiogram is often not available, studies have also used the unaffected contralateral ear for comparison [1, 5, 11, 16, 20, 21] or age- and sex-related normative hearing [4, 9].

A Microsoft Excel file was developed to provide an easy-to-use tool for classifying patients to be included in clinical trials. This tool allows for the comparison of audiograms with the ISO 7029 norm [7] and automatically calculates the severity of absolute and incident-related hearing loss. The tool provides an inclusion decision, based on the predefined audiological criteria.

## Methods

**Figure 1:**
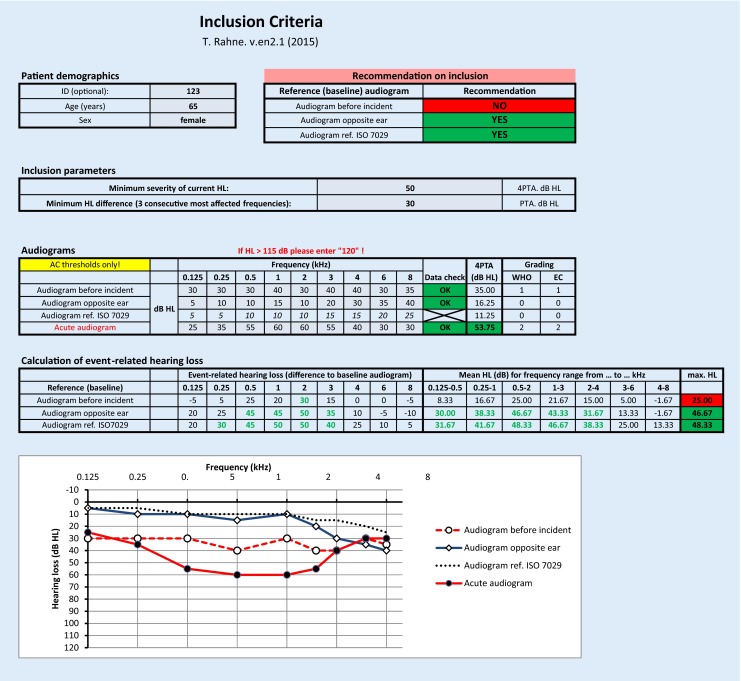

A Microsoft Excel 2010 (Version 14.0. 7151.5001; Microsoft, Redmond, Wash.) spreadsheet was developed. The main user interface with several tables and a chart are shown in Fig. 1. The user enters the subject-related parameters into the blue-shaded fields. The “patient demographic” table contains the optional ID, age, and sex of the patients. The user can modify the preset inclusion criteria for the respective clinical trial in the “inclusion parameters” table. The “minimum severity of hearing loss” is defined as the minimum hearing level (4PTA; 0.5, 1, 2, 4 kHz) of the acute audiogram needed for inclusion into the study. The “minimum hearing level” difference is calculated as the maximum difference between the mean hearing thresholds of three consecutive frequencies of the acute audiogram and the respective baseline. If the respective inclusion criteria do not have to be used, the value has to be set to zero.

**Figure 2:**
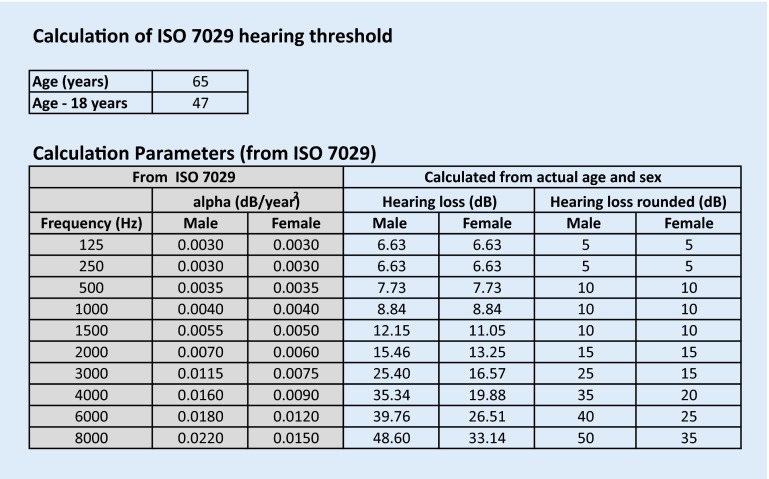

The hearing thresholds of the acute and the baseline audiograms are entered in the “audiograms” table. Thresholds above 115 dB HL are “dummy coded” with 120 dB HL, as suggested previously [2, 13]. If enough valid data points have been entered, the color of the “data check” field turns from red to green. The color of the 4PTA fields changes to green if the value is higher than the predefined minimum severity of current hearing loss. To calculate the normal age- and sex-related audiogram for every frequency, the median hearing threshold is calculated as requested by the ISO 7029 norm (see Fig. 2: worksheet “ISO 7029” of the software tool).

**Figure 3:**
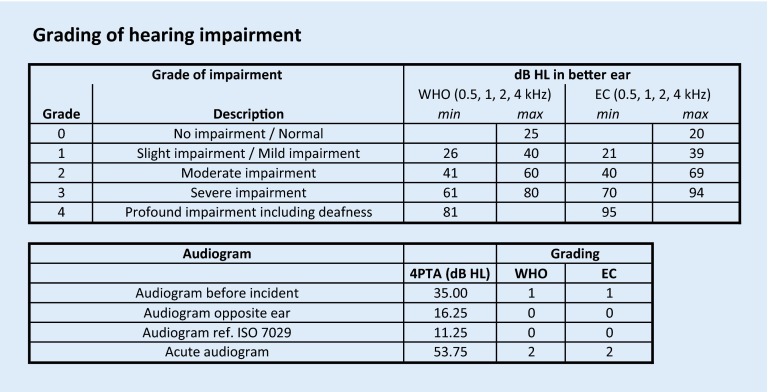

Grading of hearing impairment is displayed for every acute or baseline audiogram, assuming it is the better ear. The WHO and European Commission scores are displayed based on the data of the “grading” worksheet (Fig. 3). Both grading scores are based on the 4PTA. Various other classification systems [3, 17] can be applied by changing those data. As the affected ear may often be the worse hearing ear, the grading calculation is for qualitative information only.

The “recommendation on inclusion” is based on the inclusion parameters, separately for every baseline audiogram. If both the severity of hearing loss and the hearing loss of three consecutive frequencies meet the predefined inclusion criteria, a positive decision is recommended and the color of the fields change from red to green. A chart of the baseline and acute audiograms is provided below the tables to provide a visual impression of the plausibility of the entered data.

The applicability and the efficiency of the tool was evaluated by classifying audiograms according to the criteria of a planned, controlled, three-armed, multicenter, randomized, triple-blind study on the efficacy and safety of high-dose glucocorticosteroid treatment of ISSHL. Retrospectively, audiograms of 100 patients with ISSHL were classified to meet the respective inclusion criteria and the time needed for manual classification was compared with automatic classification using the tool.

## Results

The data of a 65-year-old female candidate are shown in Fig. 1. Pure-tone audiograms were available for the affected ear before and after the incident (i. e., a sudden hearing loss). The severity of the hearing loss was 53.75 dB HL and, thus, above the predefined value of 50 dB HL. Grading of hearing impairment (for the affected ear) was 2, which is “moderate impairment”.

The comparison with the baseline audiogram of the affected ear before the hearing loss shows only one frequency (2 kHz) with an incident-related difference above the inclusion criteria (30 dB). Thus, three consecutive frequencies with incident-related hearing loss could not be found. When compared with the audiogram of the opposite, non-affected ear, three consecutive frequencies with hearing change due to the incident ≥30 dB could be found in the frequency ranges of 0.125–0.5 kHz, 0.25–1 kHz, 0.5–2 kHz, 1–3 kHz, and 2–4 kHz. The maximum mean difference in three consecutive frequencies was in the range of 0.5–2 kHz (46.67 dB). Thus, the predefined audiological inclusion criteria were met. If (1) no previous audiogram of the affected ear is available and if (2) according to the patient’s medical history hearing was symmetric before the incident, the patient would be recommended for inclusion into the clinical trial.

The normal age- and sex-related audiogram, according to ISO 7029, was calculated and showed slightly better values than the audiogram of the opposite ear. Thus, the maximum mean difference in three consecutive frequencies was in the range of 0.5–2 kHz (48.33 dB), which is above the predefined criteria of ≥30 dB. In this case, if (1) no previous audiogram of the affected ear was available, and (2) according to the patient’s medical history the hearing was not symmetric before the incident, and (3) there was no former incident of acute hearing loss in the affected ear (e. g., due to previous ISSHL or ear surgery, both of which are often excluded in sudden hearing loss trials), a recommendation for inclusion into the clinical trial would be made.

The mean time needed for manual classification of 100 audiograms was 87 s (SD: 30 s). When classifying automatically using the tool, the mean time was 57 s (SD: 14 s). The mean time reduction was 30 % (30 s per patient) and was significant [*t*(99)=11.5, *p* < 0.001]. The decision about inclusion or exclusion did not differ between the methods.

## Discussion

The software tool presented here provides an easy method to rapidly obtain information about the inclusion of patients into a clinical trial and can help the investigator or others who are involved in selecting appropriate patients for clinical trials (e. g., referring doctors) to screen patients for eligibility. The recommendation can be printed or stored as a digital file to be archived. The most important parameters can quickly be changed and the tool is easily adaptable to a variety of studies. More modifications can be made, e. g., including only a single most affected frequency, using a three-frequency average (e. g., 3PTA_0.5–2_) instead of a four-frequency average (e. g., 4PTA_0.5–4_), or using the different frequency ranges (e. g., 4PTA_0.5–3_ vs. 4PTA_0.5–4_).

We recently classified our patients with ISSHL for inclusion in a planned, controlled, three-armed, multicenter, randomized, triple-blind study on the efficacy and safety of high-dose glucocorticosteroid treatment. We could quickly measure the estimated recruitment rate (based only on audiological inclusion criteria). The time reduction necessary for classifying audiograms using the tool was significant and relevant. In addition, the tool can be used in other centers for a proposed trial. The estimated recruitment rate is expected to be more easily available or even more reliable than if the number of eligible patients is estimated on a more subjective basis. Thus, the final planning of the number of centers in a proposed trial might be improved.

## Conclusions and practical relevance

- The software tool can simplify the screening of audiograms to compare with inclusion criteria of clinical trials.
- It can be used to estimate the recruitment rate of clinical trials as well.

## Caption Electronic Supplementary Material



## Abbreviations

3PTA: Three-frequency pure-tone average
4PTA: Four-frequency pure-tone average
ISSHL: Idiopathic sudden sensorineural hearing loss
WHO: World Health Organization
